# MiR-494 acts as a tumor promoter by targeting CASP2 in non-small cell lung cancer

**DOI:** 10.1038/s41598-019-39453-2

**Published:** 2019-02-28

**Authors:** Qiao Zhang, Yan Li, Mei Zhao, Hong Lin, Wenjie Wang, Dongdong Li, Wei Cui, Caihong Zhou, Jialing Zhong, Changzhi Huang

**Affiliations:** 1 Department of Etiology and Carcinogenesis, State Key Laboratory of Molecular Oncology, Beijing Key Laboratory for Carcinogenesis and Cancer Prevention, National Cancer Center/National Clinical Research Center for Cancer/Cancer Hospital, Chinese Academy of Medical Sciences and Peking Union Medical College, Beijing, 100021 China; 2 Clinical Laboratory, Sichuan Academy of Medical Science and Sichuan Provincial People’s Hospital, School of Medicine, University of Electronic Science and Technology of China, Chengdu, 610072 China; 3 Department of Clinical Laboratory, National Cancer Center/National Clinical Research Center for Cancer/Cancer Hospital, Chinese Academy of Medical Sciences and Peking Union Medical College, Beijing, 100021 China; 4 Department of Education, National Cancer Center/National Clinical Research Center for Cancer/Cancer Hospital, Chinese Academy of Medical Sciences and Peking Union Medical College, Beijing, 100021 China

## Abstract

MiR-494 plays an important role in several types of human cancers, including non-small cell lung cancer (NSCLC). Although the role of miR-494 has been investigated in several studies, the expression profile and underlying mechanism are still poorly understood. In this study, we found that overexpression of miR-494 promoted the proliferation and colony formation of NSCLC cells and reduced their sensitivity to cisplatin-induced apoptosis. By using microarray and Dual luciferase reporter assays, we further showed that caspase-2 (CASP2) is a functional target of miR-494, and the expression of CASP2 is inversely associated with miR-494 *in vitro*. In addition, miR-494 promoted the proliferation and colony formation of NSCLC cells and reduced their sensitivity to cisplatin-induced apoptosis by targeting CASP2. Therefore, our results suggest that miR-494 plays an oncomiR role in NSCLC cells and may be a candidate biomarker for malignant transformation and a therapeutic target of NSCLC.

## Introduction

Lung cancer is a leading cause of cancer-related morbidity and mortality worldwide. Lung cancer morbidity and mortality rates are the highest in China, and NSCLC comprises the majority of cases (around 85%)^1–3^. About NSCLC, complete surgical resection is the most effective treatment for the moment. However, the overall prognosis and survival rate after surgery remains unsatisfactory. Recently, chemotherapy is a major treatment in the management of NSCLC, and platinum-based chemotherapy including cisplatin is widely used as an important alternative, especially in advanced patients^4^. However, cisplatin resistance has been a major impediment due to the continuous infusion or multiple administrations which is a great limit to successful treatment and results in further metastasis and relapse^5–7^. Therefore, more effort is needed to uncover the underlying molecular mechanisms of NSCLC and identify new therapeutic targets to improve the survival probability of NSCLC patients.

MicroRNAs (miRNAs) are part of a class of 21 to 25 nucleotides of noncoding RNAs that are known to posttranscriptionally regulate gene expression mainly through 3-untranslated region (3-UTR) binding of mRNAs. Accumulating evidence indicates that the deregulation of miRNAs has played an essential role in the development and progression of NSCLC. MiRNAs act as oncogenes and tumor suppressors by regulating the expression of key molecular cellular proliferation, growth, apoptosis, and mobility^8,9^. Recently, growing evidence has indicated that miR-494 participates in several types of human cancers. It has been reported to play an oncogenic role in glioblastoma and colorectal cancer^10,11^; however, miR-494 has also been reported to be a tumor suppressor in gastric cancer^12^, cholangiocarcinoma^13^, esophageal squamous cell carcinoma, and ovarian cancer^14–17^. In NSCLC, miR-494 plays a more complicated role. Several studies found that it promoted tumor motility and angiogenesis and induced the apoptosis of NSCLC cells, while another study found that it suppressed NSCLC cell proliferation and induced senescence^18–22^. Thus, further exploration of the function of miR-494 in NSCLC is needed.

In this study, we demonstrated that overexpression of miR-494 promoted the proliferation and colony formation of NSCLC cells and decreased their sensitivity to cisplatin-induced apoptosis by targeting CASP2.

## Results

### Up-regulation of miR-494 promotes proliferation and colony formation of NSCLC cells

**Figure 1:**
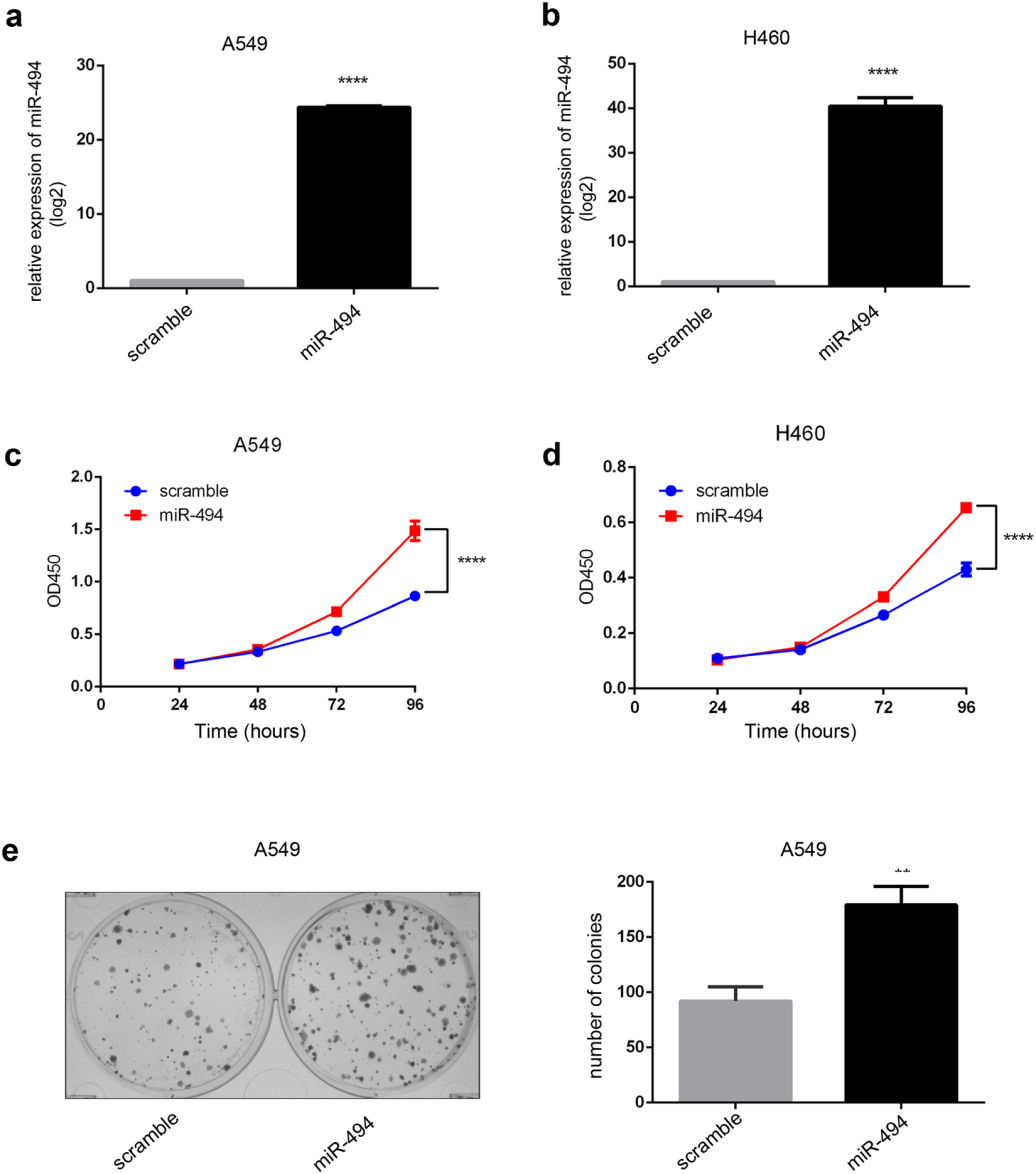
MiR-494 expression promotes cell proliferation and colony formation in NSCLC. Relative expression of miR-494 in A549 (**a**) and H460 (**b**) cell lines transfected with miR-494 mimics were validated by qRT-PCR. U6 was used as an endogenous control (****P < 0.0001). Proliferation of A549 (**c**) and H460 (**d**) with transfection of miR-494 were measured by using the CCK8 assay at indicated time points in triplicate (****P < 0.0001). (**e**) Colony formation assay was used in A549 cells treated with miR-494 or controls in triplicate (**P < 0.01).

To examine the effect of miR-494 manipulation of NSCLC cells, miR-494 mimics were transfected into A549 and H460 cells. As shown in Fig. 1a,b, cells transfected with miR-494 mimics significantly increased the expression of miR-494. Further, the increased expression of miR-494 promoted the proliferation of A549 and H460 cells (Fig. 1c,d). In addition, the effect of miR-494 overexpression was examined with regard to colony forming. We found that colony formation rates increased for A549 cells transfected with miR-494 mimics compared to the controls. These results demonstrate that the overexpression of miR-494 can increase the proliferation and colony formation of NSCLC cells.

### MiR-494 suppressed apoptosis induced by cisplatin in NSCLC cells

**Figure 2:**
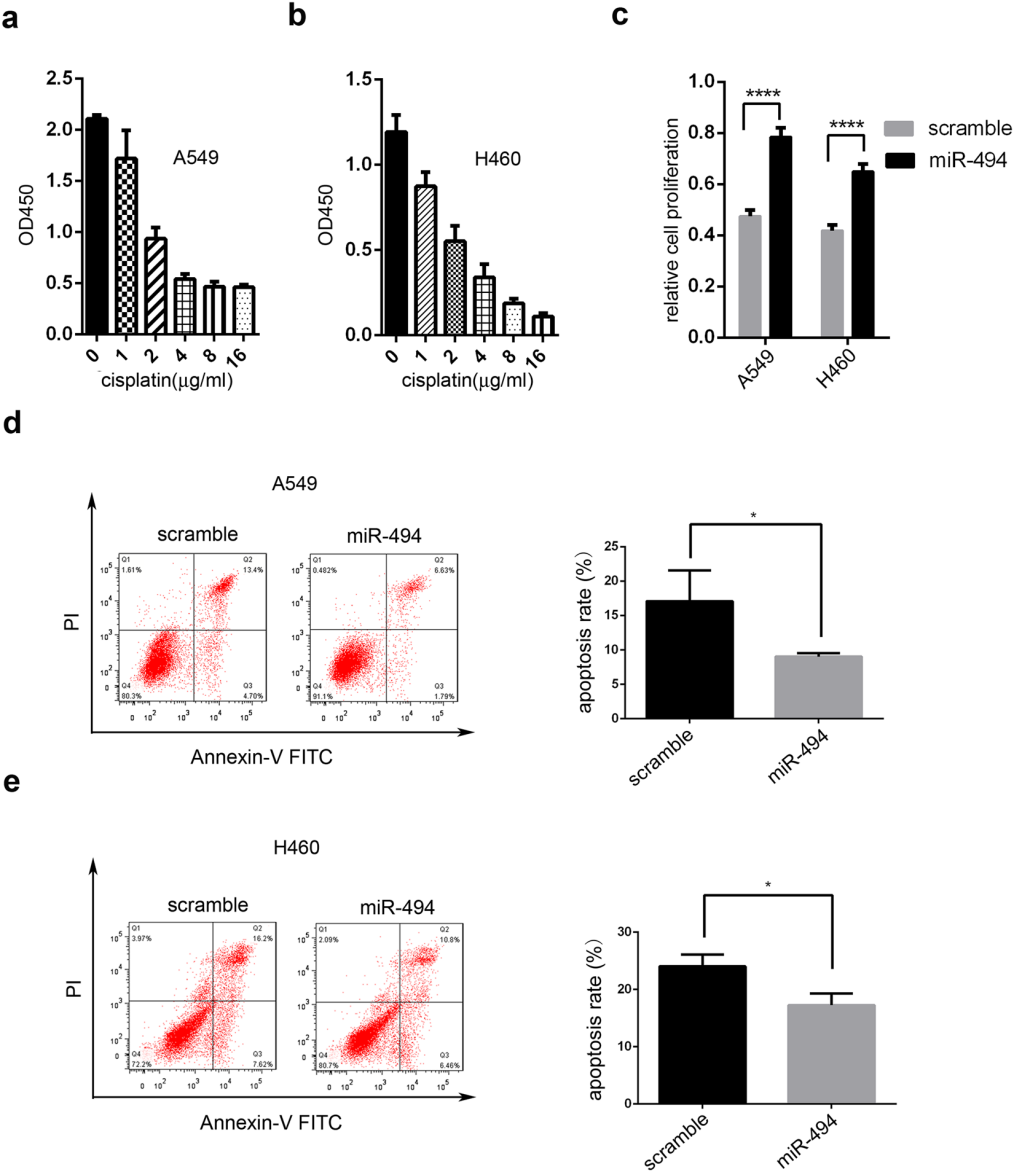
MiR-494 protects cells against apoptosis induced by cisplatin. CCK8 was used in (**a**) A549 cells and (**b**) H460 cells with different concentrations of cisplatin. (**c**) CCK8 was used in A549 and H460 cells transfected with miR-494 or controlled with cisplatin (4 mg/L) (****P < 0.0001). (**d**) A549 and (**e**) H460 cells were transfected with miR-494 mimics or controls. Cells were treated with cisplatin (4 mg/L) for 24 hours. The apoptotic cells were measured through Annexin V-FITC and Propidium Iodine staining and analyzed with FACS. The percentage of Q2 and Q3 cells was measured as the apoptosis rate. Data are presented as the ± SD of three independent experiments (*P < 0.05).

Next, we considered whether miR-494 might have the ability to affect the sensitivity of NSCLC cells to cisplatin. We used CCK8 assay to evaluate half of the maximal inhibitory concentration (IC50) for A549 and H460 cells after cisplatin gradient treatment (Fig. 2a,b). Then, we found that the overexpression of miR-494 induced an increase in cell viability in A549 and H460 cells after cisplatin treatment (Fig. 2c). Annexin-V/propidium iodide (PI) double staining was performed to study the role of miR-494 in chemoresistance. As shown in Fig. 2d,e, after treated with cisplatin, both A549 and H460 cells with miR-494 overexpression showed a significant decrease in the percentage of cell apoptosis compared to control cells.

### GO and KEGG analysis with overexpression of miR-494 in A549 cells

**Figure 3:**
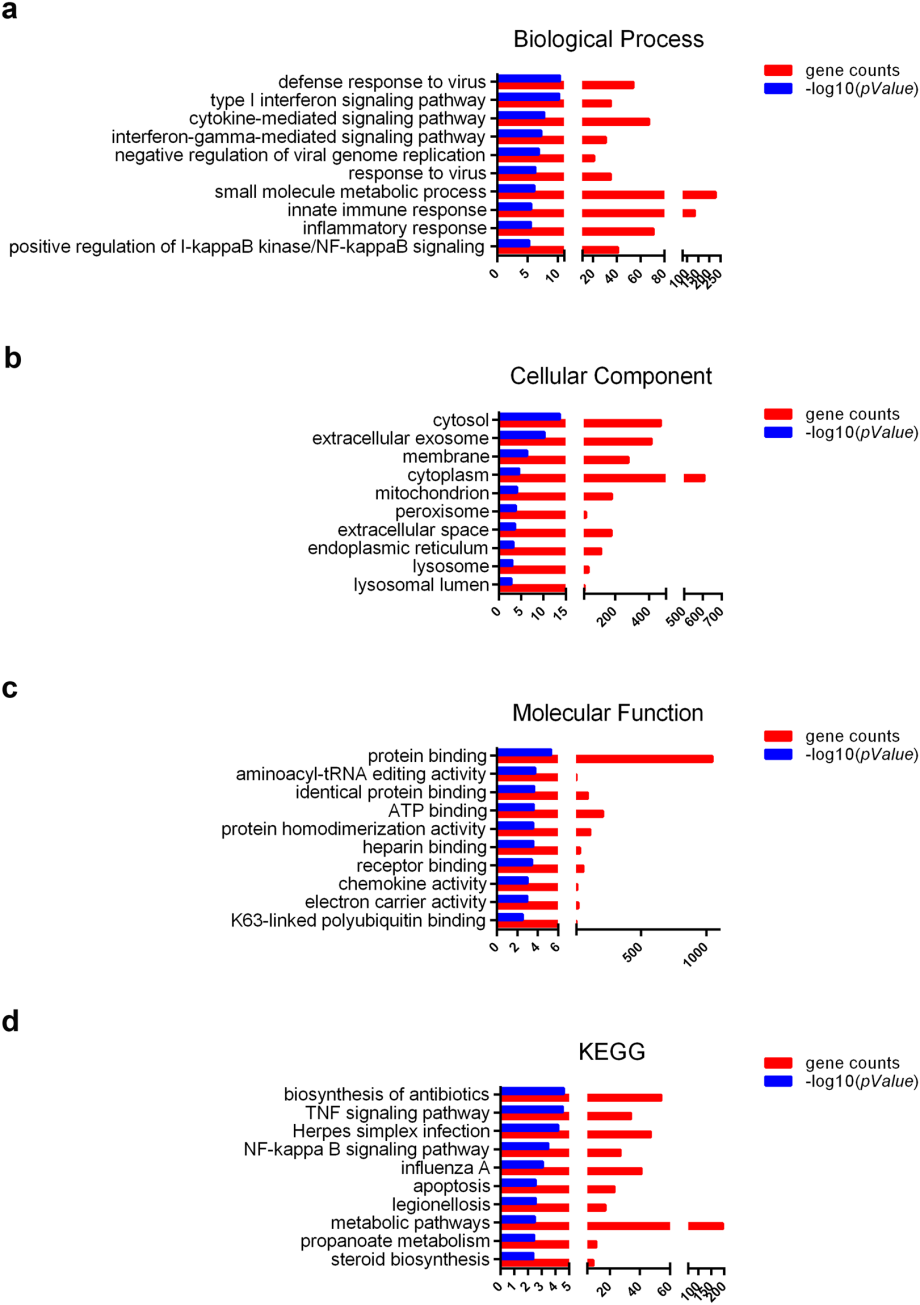
GO and KEGG analysis based on lncRNA microarray data. GO analysis, including (**a**) biological process, (**b**) cellular component, (**c**) molecular function, and (**d**) KEGG pathway analysis for differentially expressed mRNAs, was used on A549 cells transfected with miR-494 or controls.

The Agilent Human lncRNA Microarray was used after transfection with miR-494 in A549 cells. Then, GO (gene ontology) analysis (including biological process, cellular component, and molecular function) and KEGG pathway analysis were used to determine the roles of these differentially expressed mRNAs. As shown in Fig. 3, three pathways (TNF signaling pathway, NF-kappa B signaling pathway, and apoptosis pathway) were closely related to miR-494.

### CASP2 is a direct target of miR-494

**Figure 4:**
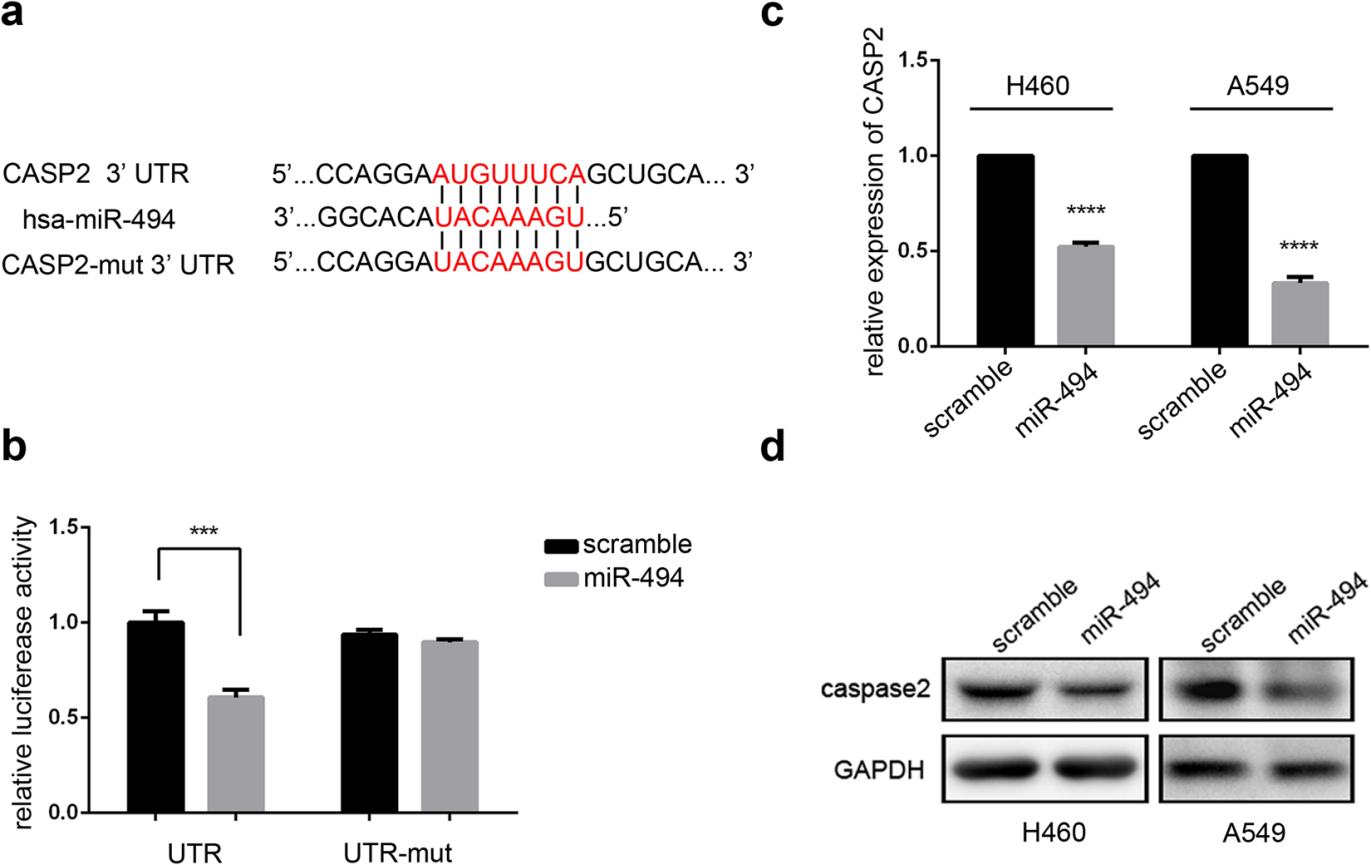
CASP2 is a target of miR-494 in human lung cancer. (**a**) MiR-494 and its binding sequence in the 3′ UTR of CASP2. Alignment of the seed regions is shown. The side of the target mutagenesis is indicated in red. (**b**) Sequences encoding wide-type or mutant fragments of the CASP2 3′ UTR were inserted into pmirGLO constructs. 293T cells were cotransfected with pmirGLO-UTR or pmirGLO-UTR-mut, plus miR-494 mimics or controls. Data are presented as the ± SD of three independent experiments (***P < 0.001). (**c**) CASP2 mRNA expression was analyzed with qRT-PCR after transfection of miR-494 mimics in A549 or H460 cells. Data are presented as ± SD (****P < 0.0001). (**d**) Up-regulation of miR-494 decreased the endogenous level of caspase-2 protein in A549 or H460 cells.

To further study the mechanism of miR-494 in NSCLC, two online miRNA binding algorithms, including TargetScan 7.1 and miRDB, were used to explore the mRNA target of miR-494. CASP2 in the apoptosis pathway was selected with a 3′ UTR region matching the seed sequences of hsa-miR-494 (Fig. 4a). Then, 3′ UTR of wide-type or mutant were respectively cloned into the pmirGLO vector downstream the firefly luciferase ORF. Renilla luciferase was used as the control for normalization. We cotransfected the reporter vector and miR-494 into 293T cells and measured the luciferase activity. We found that luciferase activity was significantly reduced in counterparts cotransfected with CASP2 wide-type and miR-494 compared to the cells transfected with scramble microRNA. Further, miR-494 or scramble microRNA cotransfected with mutant showed no effect on the activity of the reporter (Fig. 4b), which suggested that CASP2 was a direct target of miR-494. Subsequently, to confirm that miR-494 can inhibit CASP2 expression in cell lines, MiR-494 mimics were transfected into A549 and H460 cell lines; then, we observed that the up-regulation of miR-494 significantly decreased the endogenous expression of CASP2 at both the mRNA and protein levels (Fig. 4c,d). Taken together, these results show that CASP2 was a target of miR-494.

### MiR-494 promotes proliferation and colony formation by targeting CASP2

**Figure 5:**
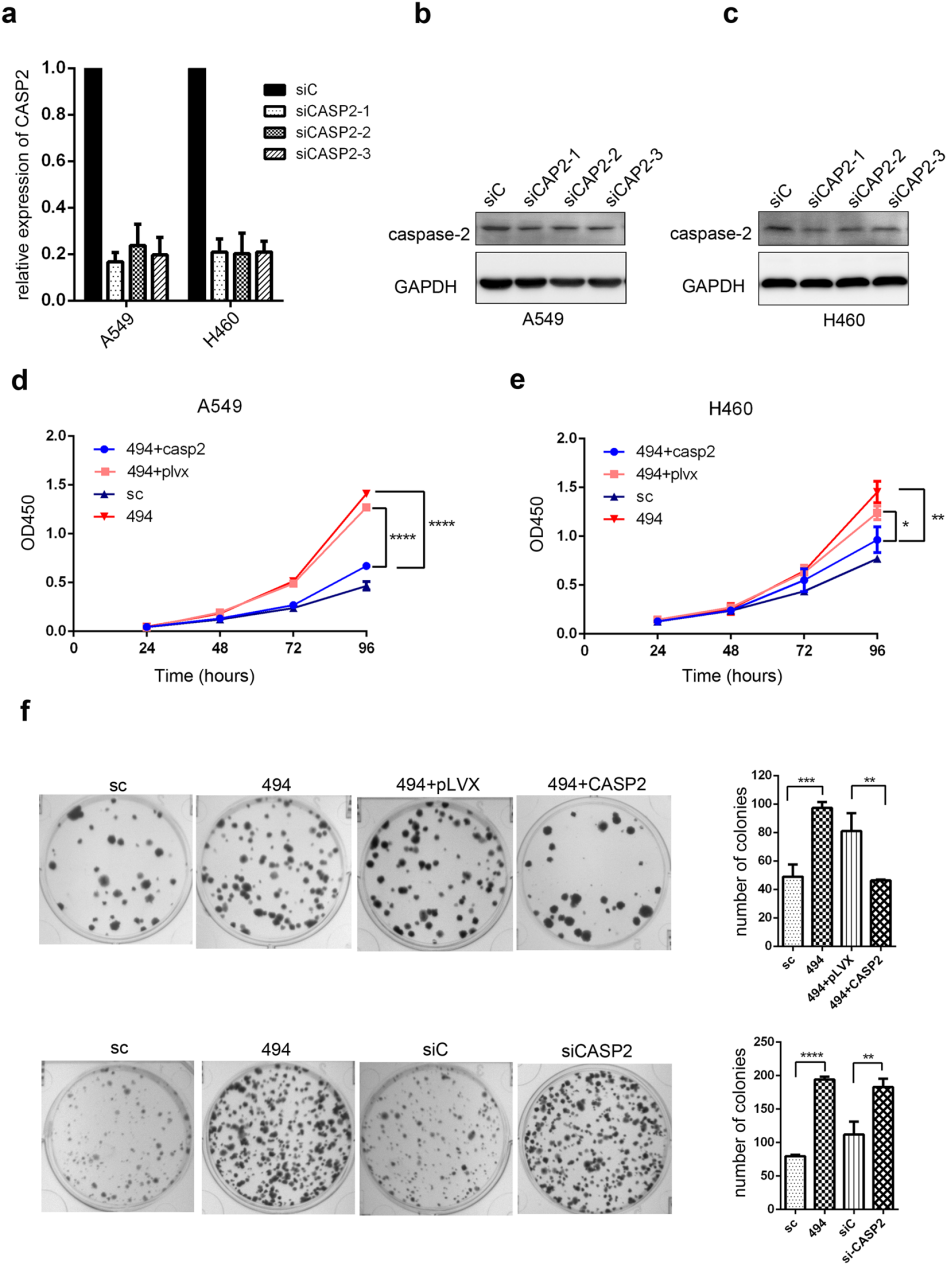
MiR-494 promotes proliferation and colony formation by targeting CASP2. (**a**) The expression levels of CASP2 were measured with qRT-PCR in A549 and H460 cells after CASP2 was knocked down by its siRNA. GAPDH was used to normalize the expression in each sample. Western blot shows relative caspase-2 expression after caspase-2 knockdown by its siRNA in (**b**) A549 and (**c**) H460 cells. Data were normalized with GAPDH. CCK8 was used for proliferation in (**d**) A549 and (**e**) H460 cells transfected with miR-494, miR-494/pLVX-CASP2-puro, or respective controls. (**f**) Colony formation assay was used in A549 cells treated with miR-494, CASP2 siRNA, miR-494/pLVX-CASP2-puro, or respective controls in triplicate (**P < 0.01, ***P < 0.001, ****P < 0.0001).

To verify that miR-494 regulates NSCLC through its target CASP2, we knocked down CASP2 with siRNA in A549 and H460 cells. As shown in Fig. 5a,c, siCASP2-1 was used in the following experiments. CCk8 analysis of miR-494/CASP2 siRNA-transfected A549 and H460 cells indicated that the up-regulation of miR-494 promoted proliferation capacity via the down-regulation of CASP2 expression (Fig. 5d,e). While exploring the functional effect of miR-494 and CASP2 on A549 cells by colony formation assay, we found that the overexpression of miR-494 or knockdown of CASP2 can promote colony formation. Furthermore, we found that the promotion of colony formation mediated by miR-494 was rescued by the overexpression of CASP2 (Fig. 5f). These results suggest that miR-494 promotes proliferation and colony formation by directly targeting CASP2 *in vitro*.

### MiR-494 reduces the sensitivity of NSCLC cells to cisplatin through targeting CASP2

**Figure 6:**
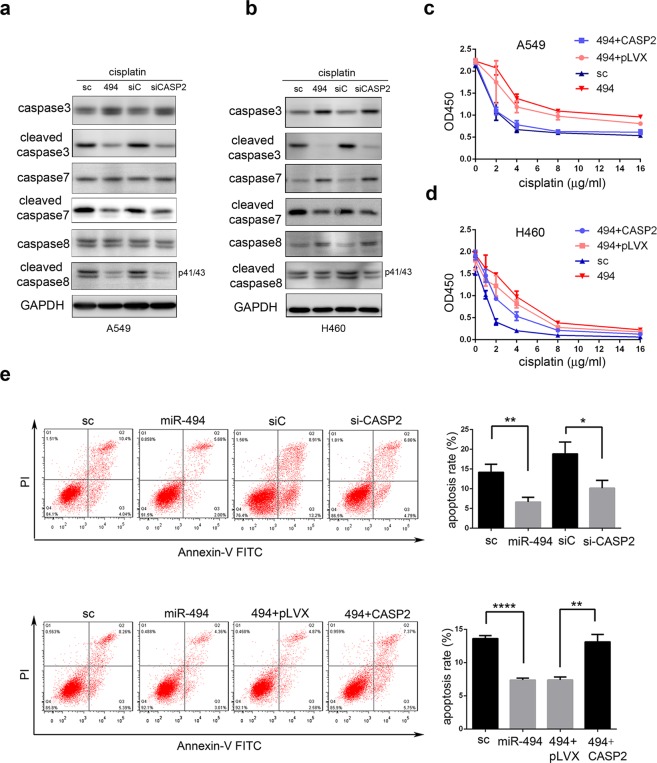
MiR-494 decreases cellular sensitivity to cisplatin through caspase-2 down-regulation in A549 cells. Western blots showing caspase-3/7/8, cleaved caspase-3/7/8 after transfection of miR-494, or siCASP2 in (**a**) A549 and (**b**) H460 cells treated with cisplatin (4 mg/L) for 24 hours. Cell viability in (**c**) A549 and (**d**) H460 cells transfected with miR-494, miR-494/pLVX-CASP2-puro, or respective controls treated with different concentrations of cisplatin for 24 hours were measured with CCK8 array. (**e**) Apoptosis rate was shown in A549 cells transfected with miR-494, siCASP2, miR-494/pLVX-CASP2-puro, or respective controls through flow cytometry after treatment with cisplatin (4 mg/L) for 24 hours. The apoptotic cells were measured through Annexin V-FITC and propidium iodide staining and analyzed with FACS. The percentage of Q2 and Q3 cells was measured as apoptosis rate. Data are presented as the ± SD of three independent experiments. *P < 0.05; **P < 0.01; ****P < 0.0001.

To investigate whether miR-494 reduces the sensitivity of NSCLC cells to cisplatin through targeting CASP2, western blot analysis was used in A549 and H460 cells. The overexpression of miR-494 or knockdown of CASP2 led to a reduction in caspase3, caspase7, and caspase8 cleavage after cisplatin treatment (Fig. 6a,b). From CCK8 assay after treatment with different concentrations of cisplatin, we found that the enhancement of cell viability mediated by miR-494 was rescued by the overexpression of CASP2 in A549 and H460 cells (Fig. 6c,d). Furthermore, we found that both the overexpression of miR-494 and knockdown of CASP2 could decrease the apoptosis rate in A549 cells. Subsequently, we confirmed that the inhibition of apoptosis mediated by miR-494 was rescued by the overexpression of CASP2 (Fig. 6e). Collectively, these results demonstrate that miR-494 is an important regulation factor for sensitivity in chemotherapies and biotherapies through targeting CASP2.

## Discussion

Human miR-494 maps chromosome 14q32.31 and was initially identified as one of the up-regulated miRNAs in human retinoblastoma tissues and Waldenström macroglobulinemia cells^23^. In NSCLC, miR-494 has been found to be overexpressed in cancer tissues associated with lymph node metastasis, tumor differentiation, tumor stages, and worsened prognoses^21,22^. Although much evidence indicates that miR-494 is related to NSCLC cell proliferation, the exact effect remains ambiguous. Some investigations have shown that miR-494 promotes A549 and H460 proliferation and clonogenic capacity *in vitro* and *in vivo*^19,22^, whereas another investigation indicated that miR-494 suppresses A549 proliferation and colony-forming activity^20^. In this study, by transferring miR-494 mimics into A549 and H460 cells, we confirmed the positive role of miR-494 in cell proliferation and the clonogenic capacity of NSCLC cells. Previous studies also indicated that miR-494 is involved in inducing cell chemoresistance. It increases hepatocellular carcinoma cell resistance to sorafenib but sensitizes colon cancer cells to fluorouracil^24,25^. In our research, we transfected miR-494 mimics into A549 and H460 cells treated with cisplatin, and we found that it suppressed cell apoptosis induced by cisplatin. These data support miR-494’s oncomiR role in NSCLC cells.

Further investigation was carried out to identify the underlying molecular mechanism of miR-494’s oncomiR role in NSCLC. GO, KEGG pathway analysis, TargetScan 7.1, and miRDB were used to explore the mRNA target of miR-494, and CASP2 was selected. CASP2 is a member of the cysteine protease family. In recent years, experimental evidence has indicated that CASP2 acts as a tumor suppressor^26,27^. It is associated with the deregulation of cell proliferation since caspase-2-deficient tumors from mice have been shown to display an increased proliferation rate. Further, it is also correlated with chemotherapeutic drug resistance since caspase-2-deficient oocytes are resistant to apoptosis induced by chemotherapeutic drugs. Moreover, relative deficits in procaspase-2 expression levels may contribute to cellular prednisolone, vincristine, and L-asparaginase (PVA) resistance in childhood acute leukemia^28^.

Using dual luciferase reporter assays, we confirmed that CASP2 was a direct target of miR-494. The overexpression of miR-494 significantly decreased the endogenous expression of CASP2 at the mRNA and protein levels. Through proliferation and colony formation assays, our study confirmed that NSCLC growth was promoted by miR-494, and this promotion could be rescued by CASP2. Since the overexpression of miR-494 significantly enhanced the proliferation capacity of cisplatin treated in A549 cells, and the enhancement was rescued with CASP2, accompanied by the lower expression of cleaved caspase3, cleaved caspase8, and cleaved caspase9, we speculated that these proliferations may be due to the resistance of cisplatin-induced apoptosis. Consistent with our speculation, the overexpression of miR-494 or knockdown of CASP2 decreased the apoptosis rate of cisplatin-treated A549 cells. Further, in the rescue experiment, CASP2 overexpression rescued the effect of miR-494 on cisplatin-treated A549 cells, indicating that miR-494 reduces NSCLC cells’ sensitivity to cisplatin-induced apoptosis by targeting CASP2.

In summary, we confirmed that miR-494 promoted the proliferation and colony formation of NSCLC cells and decrease cisplatin-induced apoptosis by targeting CASP2. Therefore, miR-494 plays an oncomiR role in NSCLC cells and may be a candidate biomarker for malignant transformation and a therapeutic target of NSCLC.

## Materials and Methods

### Cell culture

A549 and 293T cells were seeded and cultured in Dulbecco’s Modified Eagle Media (DMEM) and H460 cells in RPMI-1640 medium. All of the cell lines were maintained with 10% FBS, 100 IU/ml penicillin, and 100 IU/ml streptomycin in a 5% CO2 humidified environment at 37 °C.

### Microarray data

For the gene expression profile in A549 cells with overexpressed miR-494 or controlled miRNA, the Agilent Human lncRNA Microarray V6 (4*180K, Design ID: 084410) (Agilent Technologies, Santa Clara, CA, USA) was used in the experiment. The threshold set for up- and down-regulated genes was a fold change ≥2.0.

### RNA extraction and quantitative RT-PCR

We used Trizol (Invitrogen, USA) regent to isolate total RNA from cultured cells according to the manufacturer’s protocol; 2 µg of total RNA were reverse transcribed with random primer. Reactions contained 4 µl of 5 X buffer, 1 µl of 10 mmol/L (mM) dNTP, and 0.5 µl of reverse transcriptase (TaKaRa, Japan); DEPC water was added up to a total volume of 20 µl. Primer, DEPC water, and RNA were first incubated at 70 °C for 10 minutes, followed by dNTP, buffer, reverse transcriptase at 30 °C for 10 minutes, 42 °C for 60 minutes, and 70 °C for 10 minutes.

Data were analyzed by the ABI 7500 Real-Time PCR Detection System (Applied Biosystems, USA) using the SYBR Premix Ex Taq II kit (TaKaRa, Japan) according to the manufacturer’s instructions. Each sample was performed in triplicate, and melt curve was confirmed for the specificity of each reaction. Expression levels of miRNAs were normalized using U6 as an internal reference through the −ΔΔct method. GAPDH was used for normalizing the expression levels of mRNAs with the 2^−ΔΔct^ method.

### Transfection

Transfection for has-miR-494-3p mimics (RiboBio, Guangzhou) and CASP2 RNAi (Viewsolid Biotech, China) was carried out using Lipofectamine RNAiMAX reagent (Invitrogen, USA) with nonhomologous oligopeptides as the negative control. We used Lipofectamine 2000 (Invitrogen, USA) for the transfection of plasmids according to the manufacturer’s protocol.

### Dual luciferase reporter assays

To quantitatively evaluate miR-494 activity, 3, untranslated regions (UTR) of human CASP2, including regions from 1 to 500 base-pairs, were amplified through PCR and cloned downstream of the luciferase gene in the pmirGLO vector to be wild-type plasmids. The Renilla luciferase gene in the vector acted as a control reporter for normalization. To construct the mutant plasmid of CASP2, eight complementary nucleotides (5,-ATGTTTCA-3,) of CASP2 3′ UTR were introduced into the seed region using two-step PCR. In the HEK293T cells, wide-type or mutant CASP2 was cotransfected in 24-well plates with miR-494 mimics for 24 hours. Luciferase activities were detected using the Dual Luciferase Assay System (Promega, USA) according to the manufacturer’s instructions.

### Plate clone formation assay

A total of 500 cells transfected with miR-494, miR-494/pLVX-puro-CASP2, CASP2 siRNA, or respective controls in A549 cell lines were seeded into six-well plates in triplicate; 0.5% crystal violet was used to stain cells when the visually visible cell clones appeared in the culture dish and colonies were counted.

### Cell proliferation and death assay

Cell counting Kit-8 Assay (CCK8, Yeasen, China) was used for these experiments. Briefly, miR-494, miR-494/pLVX-CASP2-puro, or respective controls were transfected to A549 cell lines on six-well plates and cultured for 48 hours.

For the proliferation assay, after trypsinization, 2,000 cells were seeded into 96-well plates. OD450 was detected at 12, 24, 48, and 96 hours by adding 10 µl of CCK8 reagent. Then, for the cell death assay, 6,000 cells were seeded into 96-well plates with 0, 1, 2, 4, 8, or 16 mg/L cisplatin. After 24 hours, cell viability was measured using 10 µl of CCK8 in each well, incubating for 1.5 hours at 37 °C, followed by reading the absorbance at 450 nm. Each individual experiment was conducted in triplicate.

### Western blot analysis

A549 cells were harvested using protein lysis buffer (50 mM Tris-HCl, PH7.4; 1% Triton X-100; 150 mM NaCl; 1 mM PMSF; fresh proteinase inhibitor). Polyvinylidene fluoride (PVDF) membranes with transfected protein were blocked in 5% nonfat dry milk for 1 hour and then incubated with the primary antibody GAPDH (1: 3,000, Cell Signaling Technology) or caspase-2 (1: 1,000, Cell Signaling Technology) overnight, followed by secondary antibody incubation. The ECL chemiluminescence system was used to detect the signals (GE- LAS-4000, USA).

### Flow cytometry

After being transfected and subsequently incubated with cisplatin for 24 hours, cell apoptosis was detected with the Annexin V-FITC/PI apoptosis kit (MultiSciences Biotech, China) according to the manufacturer’s instructions. Briefly, PBS was used to wash A549 cells before being digested by trypsin without EDTA (Solarbio, China); then, cells were resuspended in 500 µl binding buffer. Samples were analyzed using the FACSCalibur flow cytometer (BD Biosciences, USA) after staining with Annexin V-FITC and PI solution for 30 minutes in the dark.

### Statistical analysis

Statistical analysis was performed using GraphPad Prism software (GraphPad Software Inc., San Diego, USA). Student’s t-test was used to distinguish differences between groups. The relationship between miR-494 expression level and clinical parameters was calculated by the Fisher’s exact test. A *P*-value < 0.05 was considered statistically significant.

## Supplementary information

supplementary information

## Data Availability

All data during this study are included in this published article.
