# Supplementary material for: MiR-494 acts as a tumor promoter by targeting CASP2 in non-small cell lung cancer
Source: Sci Rep. 2019 Feb 28;9:3008. doi: 10.1038/s41598-019-39453-2 (PMC6395740; doi:10.1038/s41598-019-39453-2)
Supplement: Supplementary file 1 — supplementary information [file 41598_2019_39453_MOESM1_ESM.pdf]

MiR-494 acts as a tumor promoter by targeting CASP2 in non-small cell lung cancer

Qiao Zhang[1], Yan Li[1], Mei Zhao[1], Hong Lin[1], Wenjie Wang[1], Dongdong Li[1], Wei Cui[2], Caihong Zhou[3], Jialing Zhong[4*] & Changzhi Huang[1*]

[1]Department of Etiology and Carcinogenesis, State Key Laboratory of Molecular Oncology, Beijing Key Laboratory for Carcinogenesis and Cancer Prevention, National Cancer Center/National Clinical Research Center for Cancer/Cancer Hospital, Chinese Academy of Medical Sciences and Peking Union Medical College, Beijing, 100021, China

[2]Department of Clinical Laboratory, National Cancer Center/National Clinical Research Center for Cancer/Cancer Hospital, Chinese Academy of Medical Sciences and Peking Union Medical College, Beijing, 100021, China

[3]Department of Education, National Cancer Center/National Clinical Research Center for Cancer/Cancer Hospital, Chinese Academy of Medical Sciences and Peking Union Medical College, Beijing, 100021, China

[4]Department of Laboratory Medicine, Sichuan Academy of Medical Science and Sichuan Provincial People's Hospital, Chengdu, 610072, Sichuan Province, China

Address correspondence to: Changzhi Huang, E-mail: huangpumc@163.com, huangcz@cicams.ac.cn or Jialing Zhong, E-mail: zhongjl03@163.com.

Supplementary data

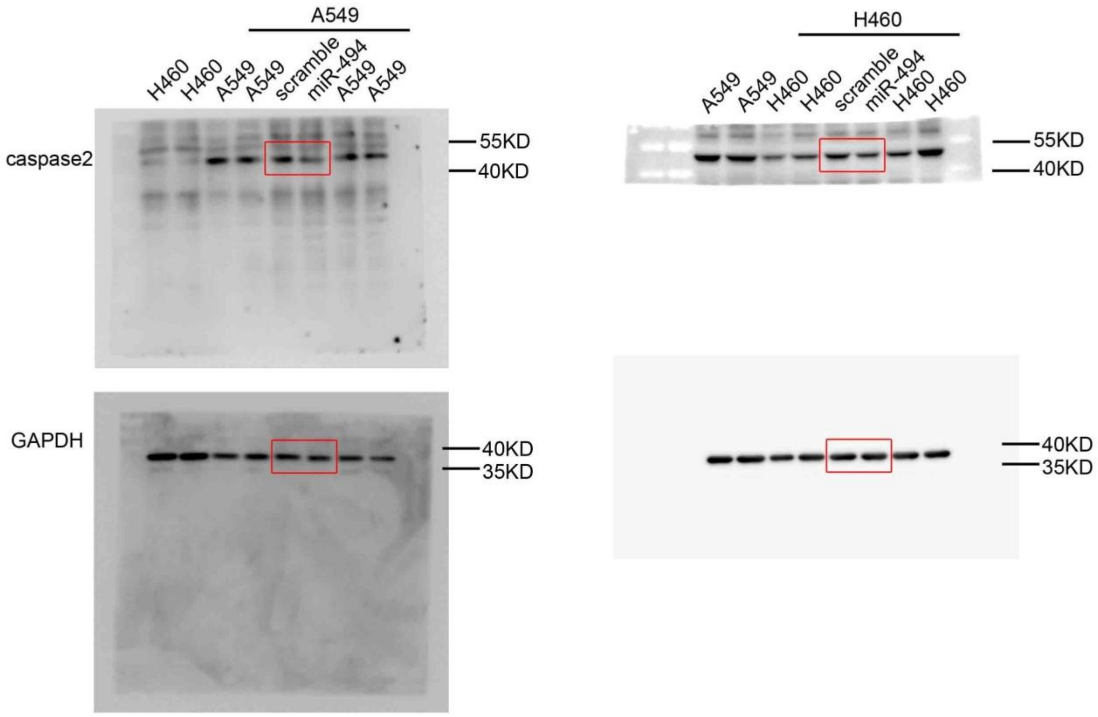

Supplemental Fig 1 The whole PVDF membrane stainings for the western blot data in Figure **5d** were presented above. Images were cropped as the indication of red lines.

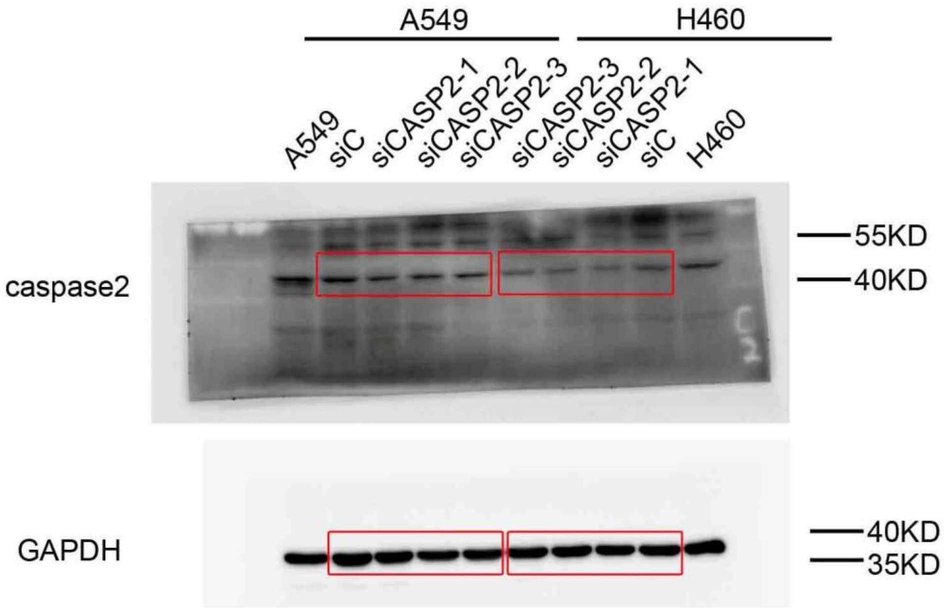

Supplemental Fig 2 The whole PVDF membrane stainings for the western blot data in Figure **6b and 6c** were presented above. Images were cropped as the indication of red lines.

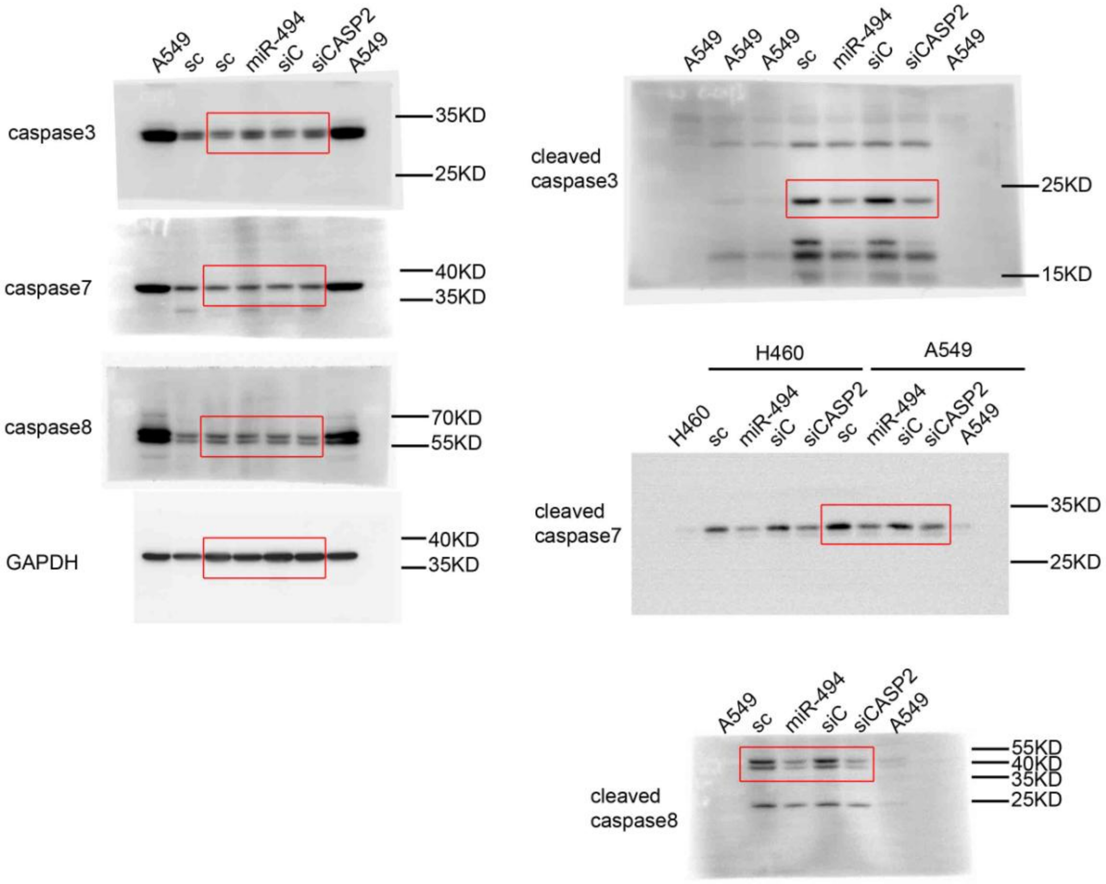

Supplemental Fig 3 The whole PVDF membrane stainings for the western blot data in Figure **7a** were presented above. Images were cropped as the indication of red lines.

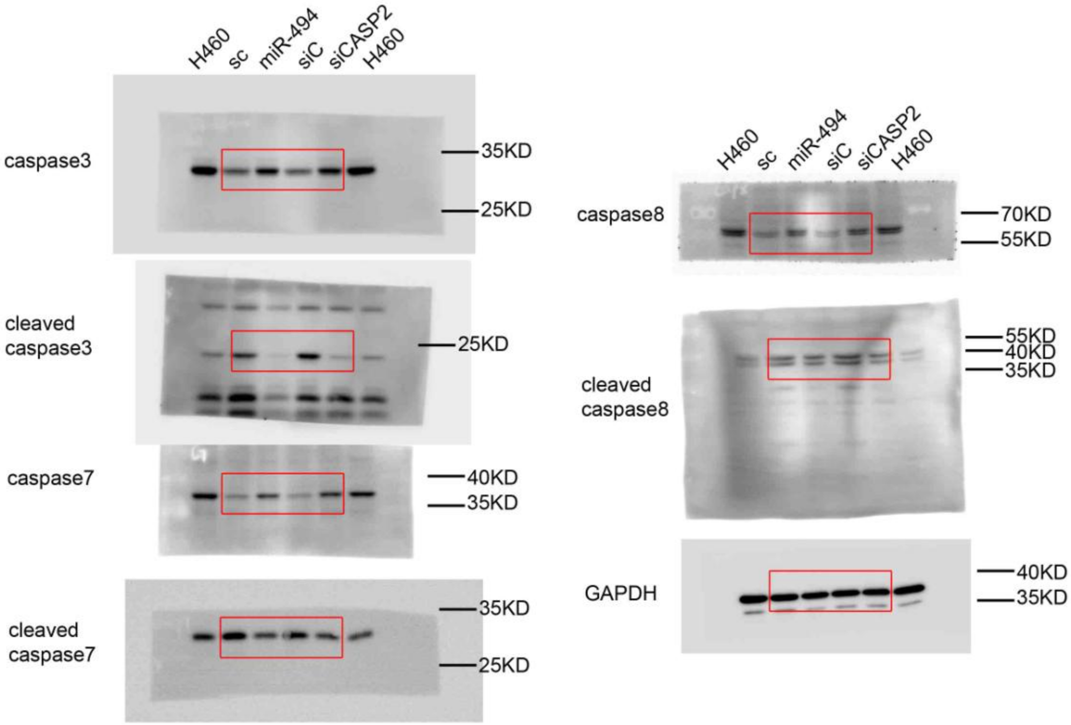

Supplemental Fig 4 The whole PVDF membrane stainings for the western blot data in Figure **7b** were presented above. Images were cropped as the indication of red lines.
